# Supplementary material for: Hot spot prediction in protein-protein interactions by an ensemble system
Source: BMC Syst Biol. 2018 Dec 31;12(Suppl 9):132. doi: 10.1186/s12918-018-0665-8 (PMC6311905; doi:10.1186/s12918-018-0665-8)
Supplement: Supplementary file 2 — All relevant MATLAB code. A simple Matlab implement of our predictor. (ZIP 19,742 kb) [file 12918_2018_665_MOESM2_ESM.zip › Additional file 2/Read ASA/ASA website/dbMPIKT/3BDYL.html]

 NetSurfP - Protein Surface Accessibility and Secondary Structure Predictions

  

|  |  |  |
| --- | --- | --- |
|  |  | NetSurfP - Protein Surface Accessibility and Secondary Structure Predictions  Technical University of Denmark |

  


---

```
# For publication of results, please cite:
# A generic method for assignment of reliability scores applied to solvent accessibility predictions.
# Bent Petersen, Thomas Nordahl Petersen, Pernille Andersen, Morten Nielsen and Claus Lundegaard
# BMC Structural Biology 2009, 9:51 doi:10.1186/1472-6807-9-51
#
# Column 1: Class assignment - B for buried or E for Exposed - Threshold: 25% exposure, but not based on RSA
# Column 2: Amino acid
# Column 3: Sequence name
# Column 4: Amino acid number
# Column 5: Relative Surface Accessibility - RSA
# Column 6: Absolute Surface Accessibility
# Column 7: Z-fit score for RSA prediction
# Column 8: Probability for Alpha-Helix
# Column 9: Probability for Beta-strand
# Column 10: Probability for Coil
E D  3BDYL                  1    0.801 115.439   0.896   0.003   0.003   0.994
B I  3BDYL                  2    0.182  33.744  -0.370   0.003   0.718   0.279
E Q  3BDYL                  3    0.416  74.333   1.716   0.001   0.959   0.040
B M  3BDYL                  4    0.097  19.390   0.462   0.001   0.959   0.040
E T  3BDYL                  5    0.419  58.046   0.779   0.001   0.900   0.099
B Q  3BDYL                  6    0.136  24.290   0.464   0.004   0.514   0.481
E S  3BDYL                  7    0.317  37.188   0.229   0.004   0.138   0.858
E P  3BDYL                  8    0.485  68.892  -1.077   0.018   0.019   0.964
E S  3BDYL                  9    0.549  64.331  -1.251   0.018   0.047   0.935
B S  3BDYL                 10    0.327  38.371  -1.458   0.022   0.359   0.619
B L  3BDYL                 11    0.250  45.683  -0.568   0.021   0.756   0.223
E S  3BDYL                 12    0.482  56.502  -0.853   0.018   0.846   0.136
B A  3BDYL                 13    0.202  22.216   0.079   0.023   0.655   0.322
E S  3BDYL                 14    0.417  48.896   0.238   0.004   0.420   0.576
E V  3BDYL                 15    0.474  72.915  -0.182   0.004   0.085   0.910
E G  3BDYL                 16    0.653  51.383  -0.908   0.005   0.015   0.979
E D  3BDYL                 17    0.473  68.116   0.601   0.004   0.138   0.858
E R  3BDYL                 18    0.539 123.523   0.883   0.004   0.616   0.381
B V  3BDYL                 19    0.073  11.235  -0.010   0.001   0.900   0.099
E T  3BDYL                 20    0.446  61.916   1.543   0.001   0.959   0.040
B I  3BDYL                 21    0.053   9.879   0.286   0.001   0.959   0.040
E T  3BDYL                 22    0.319  44.287   0.840   0.001   0.900   0.099
B C  3BDYL                 23    0.025   3.496   0.403   0.001   0.900   0.099
E R  3BDYL                 24    0.352  80.585   1.098   0.001   0.900   0.099
B A  3BDYL                 25    0.168  18.558  -1.016   0.001   0.900   0.099
B S  3BDYL                 26    0.277  32.464  -0.723   0.004   0.616   0.381
E Q  3BDYL                 27    0.465  83.120  -1.885   0.021   0.279   0.699
E D  3BDYL                 28    0.463  66.704  -2.363   0.019   0.141   0.840
E I  3BDYL                 29    0.338  62.549  -0.978   0.022   0.359   0.619
E P  3BDYL                 30    0.414  58.690  -1.800   0.005   0.336   0.660
E R  3BDYL                 31    0.455 104.195  -1.316   0.004   0.197   0.799
E S  3BDYL                 32    0.388  45.438  -1.972   0.004   0.138   0.858
E I  3BDYL                 33    0.565 104.525  -2.045   0.004   0.085   0.910
E S  3BDYL                 34    0.325  38.102  -1.079   0.005   0.262   0.733
B G  3BDYL                 35    0.165  13.017  -0.451   0.004   0.616   0.381
B Y  3BDYL                 36    0.116  24.811   0.776   0.001   0.900   0.099
B V  3BDYL                 37    0.020   3.013   1.032   0.001   0.900   0.099
B A  3BDYL                 38    0.068   7.472   0.474   0.001   0.900   0.099
B W  3BDYL                 39    0.024   5.724   0.829   0.011   0.918   0.071
B Y  3BDYL                 40    0.061  12.950   0.375   0.011   0.918   0.071
B Q  3BDYL                 41    0.156  27.808   0.332   0.002   0.816   0.182
B Q  3BDYL                 42    0.178  31.719   0.187   0.004   0.514   0.481
E K  3BDYL                 43    0.449  92.318   1.475   0.004   0.085   0.910
E P  3BDYL                 44    0.533  75.590  -0.529   0.005   0.015   0.979
E G  3BDYL                 45    0.737  58.018  -2.226   0.003   0.003   0.994
E K  3BDYL                 46    0.545 112.045   0.227   0.018   0.019   0.964
E A  3BDYL                 47    0.259  28.498  -0.491   0.018   0.047   0.935
B P  3BDYL                 48    0.106  14.985  -0.506   0.020   0.205   0.775
E K  3BDYL                 49    0.287  59.139   0.359   0.004   0.616   0.381
B L  3BDYL                 50    0.115  21.020   0.160   0.002   0.816   0.182
B L  3BDYL                 51    0.046   8.423  -0.032   0.001   0.900   0.099
B I  3BDYL                 52    0.027   4.958  -0.472   0.001   0.959   0.040
B Y  3BDYL                 53    0.095  20.323  -0.375   0.001   0.900   0.099
B W  3BDYL                 54    0.142  34.199  -1.112   0.002   0.816   0.182
B G  3BDYL                 55    0.163  12.828  -1.678   0.021   0.279   0.699
B S  3BDYL                 56    0.236  27.647  -1.802   0.019   0.141   0.840
E Y  3BDYL                 57    0.517 110.526  -1.647   0.019   0.141   0.840
E L  3BDYL                 58    0.373  68.351  -0.949   0.018   0.047   0.935
E Y  3BDYL                 59    0.562 120.206  -1.954   0.018   0.047   0.935
E S  3BDYL                 60    0.651  76.285  -2.071   0.018   0.047   0.935
E G  3BDYL                 61    0.530  41.735  -2.252   0.018   0.047   0.935
E V  3BDYL                 62    0.420  64.493  -1.168   0.018   0.047   0.935
E P  3BDYL                 63    0.551  78.244  -1.157   0.004   0.085   0.910
E S  3BDYL                 64    0.489  57.264  -1.986   0.004   0.197   0.799
E R  3BDYL                 65    0.318  72.936  -0.770   0.004   0.514   0.481
B F  3BDYL                 66    0.119  23.823  -2.242   0.003   0.718   0.279
E S  3BDYL                 67    0.298  34.926  -1.515   0.002   0.816   0.182
B G  3BDYL                 68    0.266  20.903  -2.263   0.002   0.816   0.182
E S  3BDYL                 69    0.429  50.279  -1.212   0.002   0.816   0.182
E G  3BDYL                 70    0.410  32.243  -1.167   0.004   0.616   0.381
E S  3BDYL                 71    0.538  63.007  -1.854   0.005   0.262   0.733
E G  3BDYL                 72    0.622  48.951  -1.976   0.004   0.085   0.910
E T  3BDYL                 73    0.382  53.053  -1.028   0.005   0.336   0.660
E D  3BDYL                 74    0.419  60.349   0.286   0.002   0.816   0.182
B F  3BDYL                 75    0.064  12.865  -0.246   0.001   0.900   0.099
B T  3BDYL                 76    0.236  32.789  -0.009   0.001   0.900   0.099
B L  3BDYL                 77    0.043   7.837   0.086   0.001   0.900   0.099
E T  3BDYL                 78    0.266  36.964   0.515   0.002   0.816   0.182
B I  3BDYL                 79    0.066  12.136  -0.232   0.003   0.718   0.279
E S  3BDYL                 80    0.384  44.958   0.386   0.004   0.514   0.481
E S  3BDYL                 81    0.369  43.235  -1.227   0.022   0.359   0.619
E L  3BDYL                 82    0.315  57.640  -0.736   0.020   0.205   0.775
E Q  3BDYL                 83    0.450  80.334   0.259   0.020   0.205   0.775
E P  3BDYL                 84    0.377  53.567  -1.087   0.021   0.279   0.699
E E  3BDYL                 85    0.512  89.534  -1.468   0.021   0.279   0.699
E D  3BDYL                 86    0.482  69.528  -0.867   0.005   0.262   0.733
E F  3BDYL                 87    0.404  81.023  -0.066   0.005   0.336   0.660
B A  3BDYL                 88    0.200  22.051  -1.268   0.004   0.616   0.381
E T  3BDYL                 89    0.395  54.787   0.375   0.003   0.718   0.279
B Y  3BDYL                 90    0.084  17.994   0.234   0.002   0.816   0.182
B Y  3BDYL                 91    0.138  29.384   0.913   0.002   0.816   0.182
B C  3BDYL                 92    0.147  20.681  -0.795   0.003   0.718   0.279
B Q  3BDYL                 93    0.225  40.185  -0.430   0.022   0.552   0.426
B Q  3BDYL                 94    0.183  32.630  -0.692   0.021   0.451   0.528
E H  3BDYL                 95    0.376  68.376  -1.055   0.021   0.451   0.528
E Y  3BDYL                 96    0.364  77.765  -1.230   0.022   0.359   0.619
E T  3BDYL                 97    0.361  50.112  -1.243   0.021   0.279   0.699
E T  3BDYL                 98    0.367  50.847  -0.957   0.020   0.205   0.775
E P  3BDYL                 99    0.343  48.672  -1.508   0.019   0.141   0.840
E P  3BDYL                100    0.368  52.219  -1.130   0.020   0.205   0.775
B T  3BDYL                101    0.241  33.441  -1.550   0.021   0.279   0.699
E F  3BDYL                102    0.327  65.589  -1.188   0.021   0.279   0.699
E G  3BDYL                103    0.364  28.686  -1.460   0.020   0.205   0.775
E Q  3BDYL                104    0.315  56.313  -1.766   0.019   0.141   0.840
E G  3BDYL                105    0.346  27.222  -1.718   0.019   0.141   0.840
E T  3BDYL                106    0.368  51.028  -1.002   0.021   0.279   0.699
E K  3BDYL                107    0.374  77.035  -1.074   0.021   0.279   0.699
E V  3BDYL                108    0.370  56.838  -1.374   0.021   0.279   0.699
E E  3BDYL                109    0.408  71.365  -1.143   0.022   0.359   0.619
E I  3BDYL                110    0.300  55.481  -1.366   0.021   0.451   0.528
E K  3BDYL                111    0.349  71.789  -0.988   0.021   0.451   0.528
E R  3BDYL                112    0.367  83.951  -0.842   0.022   0.359   0.619
E T  3BDYL                113    0.396  54.884  -1.673   0.021   0.279   0.699
E V  3BDYL                114    0.362  55.639  -0.940   0.019   0.141   0.840
E A  3BDYL                115    0.385  42.394  -1.328   0.020   0.205   0.775
E A  3BDYL                116    0.374  41.171  -1.511   0.021   0.279   0.699
E P  3BDYL                117    0.386  54.759  -1.460   0.022   0.359   0.619
E S  3BDYL                118    0.371  43.481  -1.401   0.021   0.451   0.528
B V  3BDYL                119    0.273  41.883  -1.430   0.021   0.451   0.528
E F  3BDYL                120    0.400  80.380  -1.549   0.022   0.552   0.426
E I  3BDYL                121    0.357  66.082  -1.891   0.021   0.451   0.528
E F  3BDYL                122    0.364  73.015  -1.620   0.022   0.359   0.619
E P  3BDYL                123    0.361  51.254  -1.637   0.021   0.279   0.699
E P  3BDYL                124    0.397  56.334  -2.128   0.020   0.205   0.775
E S  3BDYL                125    0.373  43.762  -1.781   0.019   0.141   0.840
E D  3BDYL                126    0.335  48.259  -2.103   0.056   0.142   0.802
E E  3BDYL                127    0.446  77.951  -1.505   0.064   0.216   0.721
E Q  3BDYL                128    0.348  62.171  -2.056   0.020   0.205   0.775
E L  3BDYL                129    0.480  87.925  -1.694   0.019   0.141   0.840
E K  3BDYL                130    0.430  88.554  -1.177   0.019   0.141   0.840
E S  3BDYL                131    0.553  64.776  -1.498   0.004   0.138   0.858
E G  3BDYL                132    0.517  40.688  -1.258   0.004   0.197   0.799
E T  3BDYL                133    0.392  54.370  -0.418   0.005   0.336   0.660
B A  3BDYL                134    0.085   9.323  -0.216   0.003   0.718   0.279
B S  3BDYL                135    0.174  20.358   0.068   0.001   0.900   0.099
B V  3BDYL                136    0.032   4.857   0.294   0.001   0.900   0.099
B V  3BDYL                137    0.038   5.841  -0.664   0.001   0.959   0.040
B C  3BDYL                138    0.013   1.853   1.299   0.001   0.959   0.040
B L  3BDYL                139    0.089  16.369  -0.024   0.001   0.959   0.040
B L  3BDYL                140    0.022   4.083   0.225   0.001   0.900   0.099
B N  3BDYL                141    0.197  28.812   0.270   0.002   0.816   0.182
B N  3BDYL                142    0.288  42.134  -1.608   0.004   0.514   0.481
B F  3BDYL                143    0.316  63.321  -2.039   0.005   0.336   0.660
B Y  3BDYL                144    0.261  55.797  -0.781   0.004   0.197   0.799
E P  3BDYL                145    0.347  49.253  -1.873   0.004   0.138   0.858
E R  3BDYL                146    0.611 139.873  -2.481   0.004   0.085   0.910
E E  3BDYL                147    0.380  66.351  -1.004   0.005   0.336   0.660
B A  3BDYL                148    0.105  11.549  -0.167   0.003   0.718   0.279
B K  3BDYL                149    0.243  50.047   0.216   0.001   0.900   0.099
B V  3BDYL                150    0.031   4.719   0.076   0.001   0.959   0.040
B Q  3BDYL                151    0.144  25.665   0.782   0.011   0.918   0.071
B W  3BDYL                152    0.027   6.590   0.524   0.011   0.918   0.071
B K  3BDYL                153    0.202  41.572   1.349   0.001   0.959   0.040
E V  3BDYL                154    0.312  47.878   0.222   0.002   0.816   0.182
E D  3BDYL                155    0.458  66.041   0.258   0.021   0.279   0.699
E N  3BDYL                156    0.632  92.539  -0.480   0.005   0.045   0.951
E A  3BDYL                157    0.592  65.249  -0.759   0.005   0.045   0.951
E L  3BDYL                158    0.748 136.867  -1.960   0.004   0.138   0.858
E Q  3BDYL                159    0.475  84.889  -0.530   0.004   0.138   0.858
E S  3BDYL                160    0.365  42.755  -1.271   0.005   0.045   0.951
E G  3BDYL                161    0.646  50.824  -1.984   0.005   0.045   0.951
E N  3BDYL                162    0.594  86.932  -1.807   0.004   0.138   0.858
E S  3BDYL                163    0.389  45.556  -1.234   0.004   0.420   0.576
E Q  3BDYL                164    0.354  63.171  -0.194   0.004   0.616   0.381
E E  3BDYL                165    0.514  89.813  -1.586   0.022   0.552   0.426
E S  3BDYL                166    0.372  43.634  -2.149   0.021   0.279   0.699
E V  3BDYL                167    0.432  66.322  -1.326   0.021   0.279   0.699
E T  3BDYL                168    0.299  41.471  -0.490   0.021   0.279   0.699
B E  3BDYL                169    0.287  50.226  -1.576   0.022   0.359   0.619
E Q  3BDYL                170    0.563 100.516  -1.317   0.021   0.451   0.528
E D  3BDYL                171    0.445  64.081  -1.432   0.022   0.359   0.619
E S  3BDYL                172    0.339  39.731  -1.877   0.004   0.197   0.799
E K  3BDYL                173    0.645 132.676  -1.415   0.005   0.045   0.951
E D  3BDYL                174    0.759 109.444  -2.266   0.005   0.015   0.979
E S  3BDYL                175    0.411  48.216  -1.698   0.005   0.045   0.951
E T  3BDYL                176    0.331  45.951  -0.113   0.004   0.420   0.576
B Y  3BDYL                177    0.121  25.943  -1.559   0.004   0.616   0.381
B S  3BDYL                178    0.107  12.564  -1.658   0.002   0.816   0.182
B L  3BDYL                179    0.083  15.179  -0.524   0.001   0.900   0.099
B S  3BDYL                180    0.163  19.057  -0.555   0.001   0.959   0.040
B S  3BDYL                181    0.035   4.137  -1.616   0.001   0.959   0.040
B T  3BDYL                182    0.170  23.524   0.167   0.001   0.959   0.040
B L  3BDYL                183    0.037   6.738  -0.278   0.001   0.959   0.040
E T  3BDYL                184    0.318  44.107   0.802   0.001   0.900   0.099
B L  3BDYL                185    0.063  11.480   0.564   0.003   0.718   0.279
E S  3BDYL                186    0.352  41.219   1.342   0.022   0.359   0.619
E K  3BDYL                187    0.485  99.847  -0.467   0.020   0.205   0.775
E A  3BDYL                188    0.586  64.533  -1.134   0.019   0.141   0.840
E D  3BDYL                189    0.533  76.863   0.706   0.019   0.141   0.840
B Y  3BDYL                190    0.205  43.723  -0.688   0.018   0.088   0.893
E E  3BDYL                191    0.672 117.468   0.445   0.005   0.045   0.951
E K  3BDYL                192    0.647 133.026   0.576   0.005   0.045   0.951
E H  3BDYL                193    0.630 114.633  -0.380   0.005   0.045   0.951
E K  3BDYL                194    0.526 108.198   0.373   0.005   0.336   0.660
E V  3BDYL                195    0.455  69.934   1.811   0.002   0.816   0.182
B Y  3BDYL                196    0.064  13.570   1.004   0.001   0.959   0.040
E A  3BDYL                197    0.308  33.886   1.866   0.001   0.959   0.040
B C  3BDYL                198    0.022   3.117   0.862   0.001   0.959   0.040
B E  3BDYL                199    0.249  43.465   1.364   0.001   0.959   0.040
B V  3BDYL                200    0.067  10.359   0.026   0.001   0.959   0.040
E T  3BDYL                201    0.392  54.384   0.758   0.001   0.959   0.040
B H  3BDYL                202    0.140  25.430   0.408   0.002   0.816   0.182
E Q  3BDYL                203    0.610 108.910  -0.105   0.005   0.262   0.733
E G  3BDYL                204    0.587  46.205  -1.884   0.004   0.085   0.910
E L  3BDYL                205    0.373  68.315  -0.806   0.004   0.085   0.910
E S  3BDYL                206    0.604  70.836  -0.280   0.005   0.045   0.951
E S  3BDYL                207    0.550  64.448   0.267   0.018   0.088   0.893
E P  3BDYL                208    0.435  61.755   0.195   0.021   0.279   0.699
B V  3BDYL                209    0.174  26.713  -0.361   0.004   0.616   0.381
E T  3BDYL                210    0.545  75.536   0.112   0.002   0.816   0.182
E K  3BDYL                211    0.284  58.481   1.242   0.001   0.900   0.099
E S  3BDYL                212    0.433  50.724  -0.031   0.001   0.900   0.099
B F  3BDYL                213    0.094  18.946   0.986   0.002   0.816   0.182
E N  3BDYL                214    0.490  71.765   1.652   0.004   0.616   0.381
E R  3BDYL                215    0.444 101.768   0.630   0.019   0.141   0.840
E G  3BDYL                216    0.644  50.659  -1.024   0.019   0.141   0.840
E E  3BDYL                217    0.648 113.171   1.759   0.018   0.088   0.893
E C  3BDYL                218    0.455  63.910  -1.095   0.003   0.003   0.994

Explain the output.  Go back.
```

---
